# A Novel Association of Polymorphism in the *ITGA4* Gene Encoding the VLA-4 *α*4 Subunit with Increased Risk of Alzheimer's Disease

**DOI:** 10.1155/2018/7623823

**Published:** 2018-03-27

**Authors:** Vladimira Durmanova, Zuzana Parnicka, Juraj Javor, Gabriel Minarik, Lubomir Vrazda, Barbora Vaseckova, Karin Gmitterova, Maria Kralova, Jan Pecenak, Peter Filipcik, Ivana Shawkatova

**Affiliations:** ^1^Institute of Immunology, Faculty of Medicine, Comenius University in Bratislava, Bratislava, Slovakia; ^2^Department of Molecular Biology, Faculty of Natural Sciences, Comenius University in Bratislava, Bratislava, Slovakia; ^3^Care Center Centrum Memory, Bratislava, Slovakia; ^4^Psychiatry Outpatient Clinics, University Hospital and Policlinic The Brothers of Saint John of God in Bratislava, Bratislava, Slovakia; ^5^2nd Department of Neurology, Faculty of Medicine, Comenius University in Bratislava and University Hospital, Bratislava, Slovakia; ^6^Clinic of Psychiatry, Faculty of Medicine, Comenius University in Bratislava and University Hospital, Bratislava, Slovakia; ^7^Institute of Neuroimmunology, Slovak Academy of Sciences, Bratislava, Slovakia

## Abstract

Alzheimer's disease (AD) is the most prevalent cause of dementia in elderly people worldwide. Many studies support the hypothesis that the inflammation of the CNS contributes to the neurodegeneration and disease progression. The integrin molecule *α*4*β*1, also known as very late antigen 4 (VLA-4), belongs to adhesion molecules that activate the inflammatory process through the migration of immune cells into the CNS. Therefore, the objective of our study was to analyze the association between two polymorphisms located in the *ITGA4* gene encoding the *α*4 subunit of VLA-4 and the risk of AD. 104 late-onset AD patients and 206 control subjects from Slovakia were genotyped for *ITGA4* gene SNP polymorphism rs113276800 (−269C/A) and rs1143676 (+3061A/G). The same study cohorts were also genotyped for the *APOE*-*ε*4, which is a known genetic factor associated with increased risk of AD developing. *ITGA4* polymorphism analysis revealed significantly higher frequency of the +3061AG carriers in AD group compared to the controls (*P* ≤ 0.05). Following the *APOE*-*ε*4 stratification of study groups, the association remained significant only in *APOE*-*ε*4 noncarriers. Our study suggests a novel association of *ITGA4* +3061A/G polymorphism with AD and its possible contribution to the disease pathology.

## 1. Introduction

Alzheimer's disease (AD) is a chronic neurodegenerative disease characterized by progressive memory loss, confusion, and cognitive impairment. It is the cause of 60% to 70% of dementia cases. The estimates of AD prevalence range from 4.4% in persons aged 65 years to 22% at ages 90 and older [1]. Two types of AD have been defined. Early-onset AD is manifested in people under 65 years, while the much more common late-onset AD is induced in people older than 65 years. The precise cause of AD development remains unclear. The major risk factors for AD include advanced age, genetic background, chronic diseases, head injuries, family history, and other factors [2]. The histopathological hallmarks of AD are the extracellular accumulation of the amyloid-*β* peptide creating the senile plaques and the intraneuronal fibrillar aggregates composed of abnormally phosphorylated tau proteins [3–5]. Many studies reported that neuroinflammation caused by the activation of innate immune response contributes to the pathophysiology of AD [6–10]. The innate immune response in the brain is mediated by microglia and astrocytes that internalize amyloid plaques and tau proteins. Activated microglia and astrocytes release proinflammatory cytokines (IL-1, IL-6, and TNF), chemokines (CCL2, CCL3, CXCL8), reactive oxygen radicals, and proteolytic enzymes that mediate neurodegenerative process [8]. Besides innate neuroinflammation, there is growing evidence that acquired cellular response mediated by T cells also contributes to the pathogenesis of AD [11]. In patients with AD, increased T-cell infiltration of the brain tissue and enhanced peripheral T-cell responses to amyloid-*β* have been previously described [12–15]. It was observed that different types of amyloid-*β*-specific CD4^+^ T cells in mice may promote either enhanced amyloid-*β* clearance and encephalitis or reverse cognitive decline [16–18]. However, the precise role of T cells in AD pathogenesis remains unclear.

Very late antigen 4 (VLA-4) belongs to adhesive molecules that activate the inflammatory process by facilitating the migration of immune cells into the CNS. This important member of the *β*1 integrin family is composed of two chains: CD49d (alpha 4) encoded by *ITGA4* gene and CD29 (beta 1) encoded by *ITGB1*. VLA-4 is mainly expressed on T cells, and it mediates the transmigration of T cells into the tissue by binding to its ligand VCAM-1 on endothelial cells. The role of VLA-4 in genetic predisposition to chronic inflammatory diseases of CNS has been analyzed by several studies [19–21]; however, none of them has studied its influence on AD risk. Therefore, the objective of our study was to investigate the association between genetic polymorphisms located in the *ITGA4* gene on chromosome 2q31.3 and the risk of AD. Two single nucleotide polymorphisms (SNPs) in the alpha 4-subunit gene were investigated: a nonsynonymous SNP at position 3061 (rs1143676), causing an arginine (CGG) to glutamine (CAG) transversion at amino acid position 878 in exon 24 [22], and a C to A transversion at position −269 (rs113276800) in the promoter region of the gene [23]. As the −269 (C/A) polymorphism is located near the AP-2 binding sites, the AA variant may be responsible for the negative gene expression of the *α*4 subunit. A second point mutation at +3061(A/G) leads to the formation of two *α*4 subunit variants. The G variant was named *α*4-mas, and the A variant, *α*4-tex. It is hypothesized that the G variant may change the VLA-4 *α*4 subunit conformation, leading to higher binding affinity to its ligand VCAM-1 [22].

As the *ε*4 allelic variant of the apolipoprotein E gene (*APOE*) is the most significantly AD-associated genetic factor confirmed in a number of populations [24–29], we have also analyzed its presence in our study groups and included its carriage status as a possible confounding variable in *ITGA4* association analysis.

## 2. Materials and Methods

### 2.1. Study Subjects

The investigated group included 104 unrelated individuals (31 men and 73 women) meeting criteria for Alzheimer's disease according to the ICD-10 classification [30]. Montreal Cognitive Assessment (MoCA) was chosen as the screening test for cognitive impairment in this study [31]. AD patients were recruited at random via several psychiatric clinics in Slovakia. The average age at disease onset was 79.46 ± 6.16 years. Detailed parameters of the study group are summarized in Table 1.

The reference cohort in our case-control study comprised of 206 unrelated age-matched volunteers (78 men and 128 women with the mean age of 76.22 ± 6.85 years). All control subjects were without any personal or family history of AD, and they were randomly recruited from a larger population sample. The cut-off score of 26/30 for normal cognition has been assessed by MoCA test. All AD patients and controls were Caucasians of Slovak descent. Written informed consent for enrolling in the study and for personal data management was obtained from all AD patients or their legal representatives as well as from the control subjects. All the investigations were carried out in accordance with the International Ethical Guidelines and the Declaration of Helsinki.

### 2.2. Genotyping

Both patient and control DNA were extracted from whole blood by a modified salting out procedure [32]. *APOE*-*ε*4 genotyping was performed by the determination of rs429358 (C/T) and rs7412 (T/C) polymorphisms in the fourth exon using direct sequencing. Briefly, DNA was amplified using forward primer 5′-ACTGACCCCGGTGGCGGAGGAGACGCGGGC-3 and reverse primer 5′-TGTTCCACCAGGGGCCCCAGGCGCTCGCGG-3′. PCR conditions consisted of the denaturation at 95°C for 5 minutes, followed by 40 cycles of denaturation at 95°C for 1 min, annealing at 68°C for 30 sec, and elongation at 72°C for 30 sec, and final elongation at 72°C for 7 min. The PCR products were run on 2% agarose gel for 20 min and then visualized under UV light. Their size (318 bp) was confirmed using the 100 bp DNA ladder (Solis BioDyne, EU). For direct sequencing, both forward and reverse primers were used. The sequencing was performed by BigDye Terminator v3.1 ready reaction-cycle sequencing kit according to the manufacturer's recommendations (Thermo Fisher Scientific, USA). The sequence data were analyzed using Finch TV Version 1.4.0 software (Geospiza, Inc, Washington, USA). In DNA samples, three allelic variants of *APOE* known as *APOE*-*ε*2, *APOE*-*ε*3, and *APOE*-*ε*4 have been identified. The rs1143676 single nucleotide polymorphism in the *ITGA4* gene was genotyped by PCR-RFLP as described by Andreoli et al. [20]. A 241 bp PCR product flanking the polymorphic site was amplified and afterward digested with the MspI restrictase (Thermo Fisher Scientific, USA). The restriction products were run on a 2% agarose gel for 20 min, either producing an intact PCR fragment (allele A) or two fragments of 149 and 92 bp (allele G). The rs113276800 SNP was determined by PCR with sequence-specific primers (PCR-SSP) using the method described by Heymann et al. [23]. Electrophoresis was performed in a 1.5% agarose gel for 20 minutes at 10 V/cm and the gel was UV-photographed.

### 2.3. Statistical Analysis

Allele and genotype frequencies were evaluated by direct counting. Genotypes were tested for their fit to Hardy–Weinberg equilibrium using the chi-square test. The statistical significance of differences in allele, genotype, and haplotype frequencies between AD patients and controls was evaluated in codominant, dominant, recessive, and overdominant inheritance models by the standard chi-square test using the InStat statistical software (GraphPad Software, Inc., San Diego, USA). The odds ratios (OR) and 95% confidence intervals (95%CI) were calculated as well. Finally, multivariate logistic-regression analysis adjusted for gender, age, and *APOE*-*ε*4 positivity as possible influencing factors was performed by the SNPstats web software available at https://snapstat.net/snpstats/.

## 3. Results

### 3.1. Genotyping of ITGA4 Polymorphism rs113276800

Allele and genotype frequencies of the *ITGA4* −269C/A gene polymorphism (rs113276800) observed in AD patients and control group are shown in Table 2. No statistically significant differences in allele (*P* = 0.87, OR = 1.04) and genotype frequencies (*P* = 0.85, OR = 1.05) of the SNP variant at −269C/A between the two cohorts were observed. Interestingly, no homozygous AA genotype was detected in either of the groups; however, this observation is in accordance with data obtained in other populations [20, 23]. The absence of the AA variant caused a deviation from Hardy–Weinberg equilibrium in AD patients (*χ*^2^ = 5.20, *P* = 0.02) as well as in the control group (*χ*^2^ = 9.55, *P* = 0.002). Multivariate logistic regression analysis of association between *ITGA4* rs113276800 and AD adjusted for risk variant *APOE*-*ε*4, age, and gender revealed no changes in comparison with the univariate analysis (Table 2).

### 3.2. Genotyping of ITGA4 Polymorphism rs1143676

The analysis of *ITGA4* +3061A/G polymorphism (rs1143676) distribution revealed statistically significant differences between AD patient and control cohorts (Table 2). Significantly higher frequency of +3061AG genotype in the codominant genetic model (AG versus GG, *P* = 0.04, OR = 1.89), dominant genetic model (AG + GG versus AA, *P* = 0.02, OR = 1.77) and overdominant genetic model (AA + GG versus AG, *P* = 0.01, OR = 1.83) was observed in AD patients compared to healthy controls. The multivariate logistic regression analysis of *ITGA4* rs1143676 adjusted for risk variant *APOE*-*ε*4, age, and gender revealed no changes in comparison with the univariate analysis (Table 2). Significantly higher prevalence of +3061AG carriers in AD patients compared to the controls was preserved also after the adjustment (*P* = 0.04, OR = 1.68). The haplotype analysis of *ITGA4* gene polymorphisms at positions −269 and +3061 revealed no significant differences in their prevalence in AD patients and healthy controls (*P* = 0.16–0.95, Table 2). The genotype distribution of *ITGA4* +3061A/G in AD patients did not fit the Hardy–Weinberg equilibrium (*χ*^2^ = 4.29, *P* = 0.04), whereas in the control group, they conformed to HWE (*χ*^2^ = 0.14, *P* = 0.71).

### 3.3. Genotyping of ITGA4 Polymorphisms in ApoE-*ε*4-Stratified Groups

As the *APOE*-*ε*4 confers the strongest genetic risk for AD development [33], we examined the association between the abovementioned *ITGA4* gene polymorphisms and AD in subgroups stratified for the presence of *APOE*-*ε*4. Genotyping in *APOE*-*ε*4 carriers revealed no statistically significant differences in the distribution of *ITGA4* −269C/A and +3061A/G genotypes between the patient and control groups (Table 3). On the other hand, genotyping in *APOE*-*ε*4-negative individuals showed significantly higher prevalence of +3061AG genotype in AD patients compared to the controls in the codominant genetic model (AG versus GG, *P* = 0.012, OR = 2.49), dominant genetic model (AG + GG versus AA, *P* = 0.007, OR = 2.23) and overdominant model (AA + GG versus AG, *P* = 0.003, OR = 2.40) as shown in Table 4. The multivariate logistic regression analysis of *ITGA4* genotypes in the *APOE*-*ε*4-negative group adjusted for gender and age revealed no changes in comparison with the univariate analysis, and thus, the significantly higher prevalence of +3061AG carriers in AD patients compared to the controls was preserved (AA + GG versus AG, *P* = 0.008, OR = 2.26, Table 4).

## 4. Discussion

Increasing evidence suggests that neuroinflammation plays a key role in AD pathogenesis; thus, understanding the interactions between the immune system and the nervous system might be the key to prevent or delay the disease. Innate inflammation mediated by microglia and astrocytes belongs to the main causes of disease severity and progress. Misfolded and aggregated proteins bind to pattern recognition receptors on microglia and astrocytes causing the release of inflammatory mediators that contribute to neural degeneration [8]. Besides innate inflammation, there is evidence that acquired cellular response mediated by T cells also contributes to the pathogenesis of AD [11].

The migration of leukocytes across the blood-brain barrier into tissues is mediated by cell adhesion molecules (CAMs), which are responsible for the interaction between immune cells and the surrounding environment. They play roles in cell survival, activation, and migration [34]. Many studies have shown that adhesion molecules participate in the pathogenesis of chronic inflammatory diseases including neurodegenerative diseases [35]. In AD patients, the increased expression of ICAM-1, VCAM-1, and E-selectin on endothelial cells facilitating immune cell migration to the brain parenchyma was observed [36–38]. *Trans*-migrated immune cells activate the release of proinflammatory cytokines (IL-1, IL-6, and TNF), chemokines (CCL2, CCL3, and CXCL8), reactive oxygen radicals, and proteolytic enzymes that mediate the neurodegenerative process [8].

Very late antigen 4 (VLA-4) is one of the cell adhesion molecules that activate the inflammatory process through the migration of immune cells into the CNS. As gene polymorphism can influence gene function, we have analyzed two SNPs in the *ITGA4* gene (−269C/A and +3061A/G) coding for the *α*4 chain of VLA-4 integrin on T cells. According to our knowledge, the association between *ITGA4* gene polymorphism and risk of AD has not been described until now. Our analysis of the C to A transversion at position −269 in the promoter region revealed no association with AD pathogenesis. No homozygous −269 AA genotype could be observed in either cohort, causing the deviation from Hardy–Weinberg equilibrium, however this finding was also reported by other authors [20, 23]. As the −269 (C/A) polymorphism is located in the *ITGA4* promoter region near the AP-2 binding sites, the AA variant may be responsible for the negative gene expression of the *α*4 subunit [39]. Regarding the *ITGA4* A/G gene polymorphism at +3061, we have determined significantly higher frequencies of +3061AG carriers in Slovak AD patients compared to the control group. This higher prevalence in AD patients remained significant also after the adjustment for gender, age, and *APOE*-*ε*4 positivity. A point mutation at +3061 in exon 24 that causes an arginine (CGG) to glutamine (CAG) transversion leads to the formation of two *α*4 subunit variants. The G variant was named *α*4-mas, and the A variant, *α*4-tex [22]. In healthy individuals, the frequency of *α*4-tex is much higher than that of *α*4-mas as observed in many studies including our [20, 23]. Our findings in AD patients support the previous study in which the association of the *ITGA4* +3061AG genotype with MS development was described [40]. We presume that the AG variant in patients may change the VLA-4 *α*4 subunit conformation leading to higher binding affinity to its ligand VCAM-1, but this explanation needs to be proved.

As *APOE*-*ε*4 variant confers the strongest genetic risk for AD development [24–29, 33], we have analyzed the association between *ITGA4* gene polymorphisms and risk of AD in *APOE*-*ε*4-stratified cohorts as well. After the stratification of our study groups for the presence of *APOE*-*ε*4, a significant association between *ITGA4* +3061AG genotype and AD was detected only in *APOE*-*ε*4-negative individuals. This finding allows us to suggest that the +3061A/G variant may be independently related to the pathogenesis of AD. Other studies also indicate possible association between genes coding for clusterine, neurotrophin-3, brain-derived neurotrophic factor, and others and risk of AD independent of *APOE*-*ε*4 [41–44].

Our results support the role of T cells in AD pathology. Using various models of amyloid pathology, conflicting results have been reported regarding the impact of both CD4^+^ and CD8^+^ T cells, infiltrating the brain, on disease progression, suggesting both beneficial and detrimental impacts [45, 46]. It was observed that T cells that recognize A*β*1-40 peptide can prevent the formation of A*β* plaques because their presence has been detected mainly in healthy individuals. In contrast, T cells specific for A*β*1-42 are detectable in AD patients, which indicates that they may play a role during plaque formation [47, 48]. It was also found that A*β*-specific CD4^+^ Th1 cells induce the production of proinflammatory cytokines by microglial cells, whereas A*β*-specific CD4^+^ Th2 cells mediate the inhibition of cytokine production by glial cells [14]. McManus and coworkers have suggested that Th17 cells together with Th1 cells may lead to microglial activation and inflammatory changes in the brain [49]. Alternatively, hippocampal CD8^+^ T cells might also directly affect neuronal function via cytotoxic damage of neurites [50, 51]. It is possible that different stages of AD progression have distinct T-cell subpopulation profiles and that the immune cells may play contradictory roles at the early versus late AD stages.

Results of *ITGA4* genotyping may also contribute to the development of the new AD treatment. Currently, the therapeutic options are only symptomatic, but many research studies have been dedicated to the development of various immunotherapeutic strategies [52, 53]. In regard of this, the humanized monoclonal antibody binding to the *α*4 subunit of VLA-4, Natalizumab, used for the treatment of multiple sclerosis, could be tested in AD patients for its influence on disease progression.

## 5. Conclusion

This is the first study reporting a possible role of *ITGA4* gene coding for *α*4 chain of VLA-4 integrin in the genetic susceptibility to AD. We have identified a novel independent genetic association between *ITGA4* +3061A/G variant and increased risk of AD. Our data provide additional evidence to the knowledge that besides the known genetic factors like *APOE*-*ε*4, other genetic variants may be involved in the induction of late-onset AD pathology.

## Figures and Tables

**Table 1:** Characteristics of the studied groups.

Parameter	AD subjects (*n* = 104)	Controls (*n* = 206)	*P*
Gender ratio: male/female	31/73	78/128	0.16
Mean age ± SD (years)	79.46 ± 6.16	76.22 ± 6.85	<0.0001
Mean onset age ± SD (years)	77.4 ± 6.47	—	—
MoCA score	13.62 ± 5.72	28.52 ± 1.36	<0.0001
ApoE-*ε*4 positivity (yes/no)	39/65	41/165	0.0008

SD: standard deviation; MoCA: Montreal Cognitive Assessment; *P* ≤ 0.05 is considered as significant.

**Table 2:** Allele and genotypes frequencies of the *ITGA4* −269A/C and +3061A/G polymorphism in AD patients and controls.

SNP/model	Allele/genotype	AD subjects (*n* = 104)	Controls (*n* = 206)	Univariate analysis		Multivariate analysis	
				*P*	OR (95% CI)	*P*	OR (95% CI)
−269 C/A	C	170 (81.73%)	339 (82.28%)	—	—	—	—
	A	38 (18.27%)	73 (17.72%)	0.87	1.04 (0.67–1.60)	—	—

Codominant	CC	66 (63.46%)	133 (64.56%)		1.00		1.00
	CA	38 (36.54%)	73 (35.44%)	0.85	1.05 (0.64–1.71)	1.00	1.00 (0.60–1.68)
	AA	0 (00.00%)	0 (00.00%)	—	—	—	—

Dominant	CC	66 (63.46%)	133 (64.56%)		1.00		1.00
	CA + AA	38 (36.54%)	73 (35.44%)	0.85	1.05 (0.64–1.71)	1.00	1.00 (0.60–1.68)

Recessive	CC + CA	104 (100.00%)	206 (100.00%)	—	—	—	—
	AA	0 (00.00%)	0 (00.00%)	—	—	—	—

+3061 A/G	A	135 (64.90%)	294 (71.36%)	—	—	—	—
	G	73 (35.10%)	118 (28.64%)	0.10	1.35 (0.94–1.92)	—	—

Codominant	AA	39 (37.50%)	106 (51.46%)		1.00		1.00
	AG	57 (54.81%)	82 (39.80%)	**0.04** ^∗^	1.89 (1.15–3.11)	0.11	1.76 (1.04–2.97)
	GG	8 (7.70%)	18 (8.74%)	—	1.21 (0.49–3.00)	—	1.35 (0.52–3.51)

Dominant	AA	39 (37.50%)	106 (51.46%)		1.00		1.00
	AG + GG	65 (62.50%)	100 (48.54%)	**0.02** ^∗^	1.77 (1.09–2.86)	**0.04** ^∗^	1.69 (1.02–2.81)

Recessive	AA + AG	96 (92.31%)	188 (91.26%)		1.00		1.00
	GG	8 (7.69%)	18 (8.74%)	0.75	0.87 (0.37–2.07)	0.99	1.01 (0.40–2.52)

Overdominant	AA + GG	47 (45.19%	124 (60.20%)		1.00		1.00
	AG	57 (54.81%)	82 (39.80%)	**0.01** ^∗^	1.83 (1.14–2.95)	**0.04** ^∗^	1.68 (1.02–2.77)

Haplotypes	CA	58.21%	63.70%		1.00		1.00
	CG	23.52%	18.58%	0.16	1.41 (0.88–2.25)	0.2	1.39 (0.84–2.29)
	AG	11.57%	10.06%	0.44	1.29 (0.68–2.44)	0.46	1.29 (0.66–2.53)
	AA	6.70%	7.66%	0.95	0.97 (0.41–2.31)	0.76	0.87 (0.35–2.18)

Allele and genotype frequencies are presented as absolute numbers with percentages in parentheses. OR: odds ratio; CI: confidence interval. Univariate analysis is based on *χ*^2^ test. Multivariate analysis is adjusted by gender, age, and *APOE*-*ε*4 positivity. ^∗^*P* ≤ 0.05 is considered as significant.

**Table 3:** Allele and genotypes frequencies of the *ITGA4* −269A/C and +3061A/G polymorphism in *APOE*-*ε*4-positive AD patients and controls.

SNP/model	Allele/genotype	AD subjects (*n* = 39)	Controls (*n* = 41)	Univariate analysis		Multivariate analysis	
				*P*	OR (95% CI)	*P*	OR (95% CI)
−269 C/A	C	64 (82.05%)	68 (82.93%)	—	—	—	—
	A	14 (17.95%)	14 (17.07%)	0.88	1.06 (0.47–2.40)	—	—

Codominant	CC	25 (64.10%)	27 (65.85%)		1.00		1.00
	CA	14 (35.90%)	14 (34.15%)	0.87	1.08 (0.43–2.71)	0.95	0.97 (0.36–2.61)
	AA	0 (00.00%)	0 (00.00%)	—	—	—	—

Dominant	CC	25 (64.10%)	27 (65.85%)		1.00		1.00
	CA + AA	14 (35.90%)	14 (34.15%)	0.87	1.08 (0.43–2.71)	0.95	0.97 (0.36–2.61)

Recessive	CC + CA	39 (100.00%)	41 (100.00%)	—	—	—	—
	AA	0 (00.00%)	0 (00.00%)	—	—	—	—

+3061 A/G	A	53 (67.95%)	57 (69.51%)	—	—	—	—
	G	25 (32.05%)	25 (30.49%)	0.83	1.08 (0.55–2.10)	—	—

Codominant	AA	17 (43.59%)	18 (43.90%)		1.00		1.00
	AG	19 (48.72%)	21 (51.22%)	0.87	0.96 (0.39–2.38)	0.87	0.93 (0.35–2.47)
	GG	3 (7.69%)	2 (4.88%)	—	1.59 (0.24–10.70)	—	1.58 (0.22–11.39)

Dominant	AA	17 (43.59%)	18 (43.90%)		1.00		1.00
	AG + GG	22 (56.41%)	23 (56.10%)	0.98	1.01 (0.42–2.45)	0.99	0.99 (0.39–2.56)

Recessive	AA + AG	36 (92.31%)	39 (95.12%)		1.00		1.00
	GG	3 (7.69%)	2 (4.88%)	0.6	1.62 (0.26–10.29)	0.61	1.64 (0.24–11.11)

Overdominant	AA + GG	20 (51.28%)	20 (48.78%)		1.00		1.00
	AG	19 (48.72%)	21 (51.22%)	0.82	0.90 (0.38–2.17)	0.79	0.88 (0.34–2.26)

Haplotypes	CA	60.48%	62.69%		1.00		1.00
	CG	21.57%	20.23%	0.81	1.11 (0.46–2.68)	0.78	1.15 (0.45–2.93)
	AG	10.48%	10.26%	0.88	1.10 (0.32–3.77)	0.96	0.97 (0.25–3.70)
	AA	7.47%	6.82%	0.86	1.15 (0.24–5.50)	0.93	1.09 (0.19–6.11)

Allele and genotype frequencies are presented as absolute numbers with percentages in parentheses. OR: odds ratio; CI: confidence interval. Univariate analysis is based on the *χ*^2^ test. Multivariate analysis is adjusted by gender and age. *P* ≤ 0.05 is considered as significant.

**Table 4:** Allele and genotype frequencies of the *ITGA4* −269A/C and +3061A/G polymorphism in *APOE*-*ε*4-negative AD patients and controls.

SNP/model	Allele/genotype	AD subjects (*n* = 65)	Controls (*n* = 165)	Univariate analysis		Multivariate analysis	
				*P*	OR (95% CI)	*P*	OR (95% CI)
−269 C/A	C	106 (81.54%)	271 (82.12%)	—	—	—	—
	A	24 (18.46%)	59 (17.88%)	0.88	1.04 (0.62–0.76)	—	—

Codominant	CC	41 (63.08%)	106 (64.24%)		1.00		1.00
	CA	24 (36.92%)	59 (35.76%)	0.87	1.05 (0.58–1.91)	1.00	1.00 (0.54–1.85)
	AA	0 (00.00%)	0 (00.00%)	—	—	—	—

Dominant	CC	41 (63.08%)	106 (64.24%)		1.00		1.00
	CA + AA	24 (36.92%)	59 (35.76%)	0.87	1.05 (0.58–1.91)	1.00	1.00 (0.54–1.85)

Recessive	CC + CA	65 (100.00%)	165 (100.00%)	—	—	—	—
	AA	0 (00.00%)	0 (00.00%)	—	—	—	—

+3061 A/G	A	82 (63.08%)	237 (71.82%)	—	—	—	—
	G	48 (36.92%)	93 (28.18%)	0.07	1.49 (0.97–2.29)	—	—

Codominant	AA	22 (33.85%)	88 (53.33%)		1.00		1.00
	AG	38 (58.46%)	61 (36.97%)	**0.012** ^∗^	2.49 (1.34–4.62)	**0.025** ^∗^	2.37 (1.26–4.48)
	GG	5 (7.69%)	16 (9.70%)	—	1.25 (0.41–3.78)	—	1.34 (0.43–4.20)

Dominant	AA	22 (33.85%)	88 (53.33%)		1.00		1.00
	AG + GG	43 (66.15%)	77 (46.67%)	**0.007** ^∗^	2.23 (1.23–4.06)	**0.012** ^∗^	2.18 (1.18–4.03)

Recessive	AA + AG	60 (92.31%)	149 (90.30%)		1.00		1.00
	GG	5 (7.69%)	16 (9.70%)	0.63	0.78 (0.27–2.21)	0.77	0.85 (0.29–2.50)

Overdominant	AA + GG	27 (41.54%)	104 (63.03%)		1.00		1.00
	AG	38 (58.46%)	61 (36.97%)	**0.003** ^∗^	2.40 (1.34–4.31)	**0.008** ^∗^	2.26 (1.24–4.12)

Haplotypes	CA	56.88%	63.97%		1.00		1.00
	CG	24.66%	18.15%	0.14	1.55 (0.87–2.75)	0.17	1.51 (0.84–2.72)
	AG	12.26%	10.03%	0.42	1.37 (0.63–2.96)	0.40	1.40 (0.64–3.06)
	AA	6.20%	7.85%	0.87	0.91 (0.30–2.77)	0.67	0.78 (0.25–2.45)

Allele and genotype frequencies are presented as absolute numbers with percentages in parentheses. OR: odds ratio; CI: confidence interval. Univariate analysis is based on the *χ*^2^ test. Multivariate analysis is adjusted by gender and age. ^∗^*P* ≤ 0.05 is considered as significant.

## Abbreviations

AD: Alzheimer's disease
ApoE: Apolipoprotein E
CD: Cluster of designation
CI: Confidence interval
CNS: Central nervous system
E-selectin: Endothelial adhesion molecule
ICAM-1: Intercellular adhesion molecule 1
IL: Interleukin
ITGA4: Integrin alpha 4
OR: Odds ratio
PCR: Polymerase chain reaction
RFLP: Restriction fragment length polymorphism
SNP: Single-nucleotide polymorphism
SSP: Sequence specific primer
Th: T helper cells
TNF: Tumor necrosis factor
VCAM-1: Vascular cell adhesion molecule 1
VLA-4: Very late antigen 4.
